# Pleuritic chest pain from portal hypertensive gastropathy in ESRD patient with autosomal dominant polycystic kidney disease misdiagnosed as pericarditis.

**DOI:** 10.15171/jrip.2016.11

**Published:** 2016-01-30

**Authors:** Macaulay Amechi Chukwukadibia Onuigbo, Nneoma Agbasi, Jennifer Achebe, Charles Odenigbo, Fidelis Oguejiofor

**Affiliations:** ^1^Mayo Clinic College of Medicine, Rochester, USA; ^2^Department of Nephrology, Mayo Clinic Health System, Eau Claire, USA; ^3^North East London NHS Foundation Trust, UK; ^4^Aureus University School of Medicine, Oranjestad, Aruba; ^5^Department of Medicine, Nnamdi Azikiwe Teaching Hospital, Nnewi, Anambra State, Nigeria

**Keywords:** ADPKD, Chest pain, End stage renal disease, Gastropathy, Pericarditis, Portal hypertensive gastropathy

## Abstract

Portal hypertensive gastropathy (PHG) is a gastric mucosal lesion complicating portal hypertension, with higher prevalence in decompensated cirrhosis. PHG can sometimes complicate autosomal dominant polycystic kidney disease (ADPKD) due to the presence of multiple liver cysts. Besides, PHG is known to present as chest pain, with or without hematemesis. Other causes of chest pain in ADPKD include referred chest pain from progressively enlarging kidney cysts, and rare pericardial cysts. Chest pain, especially if pleuritic, in end-stage renal disease (ESRD) patients, is often ascribed to uremic pericarditis. We present recurrent pleuritic chest pain in a 24-year old ESRD patient with ADPKD that was initially misdiagnosed as uremic pericarditis. It was ultimately shown to represent symptomatic PHG with excellent therapeutic response to proton pump inhibitors.

**Implication for health policy/practice/research/medical education:**

Portal hypertensive gastropathy (PHG) is a gastric mucosal lesion complicating portal hypertension, with higher prevalence in decompensated cirrhosis. PHG can sometimes complicate autosomal dominant polycystic kidney disease (ADPKD) due to the presence of multiple liver cysts. Besides, PHG is known to present as chest pain, with or without hematemesis. Other causes of chest pain in ADPKD include referred chest pain from progressively enlarging kidney cysts, and rare pericardial cysts. Chest pain, especially if pleuritic, in end-stage renal disease (ESRD) patients, is often ascribed to uremic pericarditis. We present recurrent pleuritic chest pain in a 24-year old ESRD patient with ADPKD that was initially misdiagnosed as uremic pericarditis. It was ultimately shown to represent symptomatic PHG with excellent therapeutic response to proton pump inhibitors.

## Introduction

Portal hypertensive gastropathy (PHG) is an important cause of gastrointestinal bleeding (GIB) and represents a gastric mucosal lesion that is an uncommon cause of overt GIB but can be frequently associated with chronic blood loss and iron deficiency anemia (1,2). As its name indicates, PHG is found in patients with portal hypertension. It is seen in patients with cirrhosis and also in cases of non-cirrhotic portal hypertension. The prevalence of PHG, as mentioned in the literature, varies from 20% to more than 80%, with higher prevalence in decompensated cirrhosis as compared with patients with chronic hepatitis C and milder liver disease or those without cirrhosis (3,4). Besides, PHG can sometimes be a complication of autosomal dominant polycystic kidney disease (ADPKD) due to the presence of multiple liver cysts (1,5). Patients with ADPKD have frequently been reported to have pericarditis due to the chronic uremia caused by the kidney failure that is often associated with this condition (6). Additionally, there have been reported rare cases of chest pain secondary to pericardial cysts in patients with ADPKD (7,8). Moreover, referred chest pain associated with stretching of the kidney capsule by enlarging cysts in ADPKD has also been described (9). However, there has not been many cases reported of chest pain in ADPD that is due solely to PHG.

We herein describe an unusual case of chest pain caused by PHG in a patient with ADPKD with multiple cysts in the liver that was initially misdiagnosed as recurrent pericarditis associated with end-stage renal disease (ESRD).

## Case Presentation

**Figure 1:**
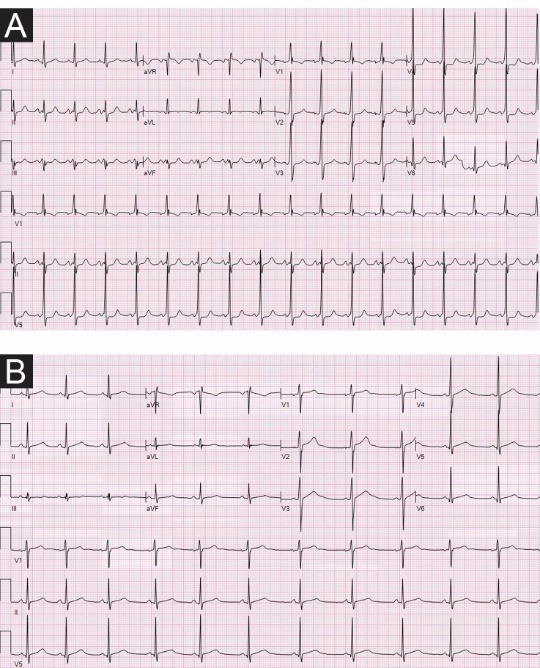
A) Showing new ST-T segment changes on EKG in mid-June 2014 that led to the misdiagnosis of acute pericarditis. B) showing normal ST-T segment changes on EKG five days later in June 2014.

**Figure 2:**
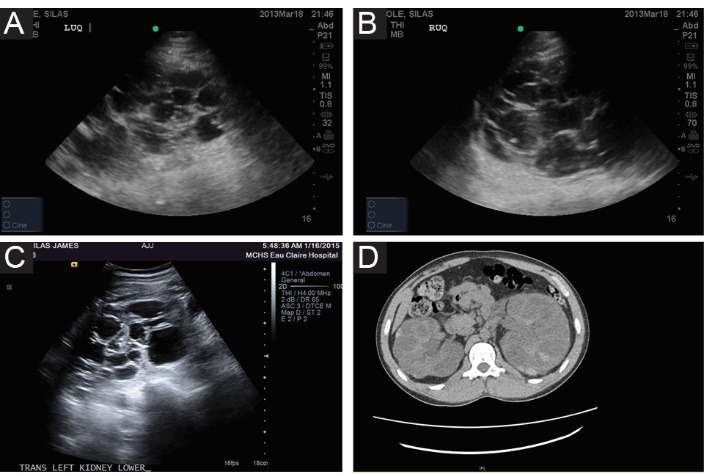
A) Left polycystic kidney in April 2013. B) Right polycystic kidney in April 2013. C) Left polycystic kidney in January 2015. D) CT scan of the polycystic kidneys in January 2013; the left is the bigger kidney.

A 24-year-old Caucasian male with a past medical history of ESRD secondary to familial ADPKD diagnosed in 2006 and who had been on maintenance hemodialysis since August 2013, hypertension, had undergone a right-sided nephrectomy of a large 22 cm sized polycystic kidney in April 2013 for intractable and life-threatening gross hematuria, intermittent left flank pain and suspected rupture of kidney cysts. Wolff-Parkinson-White Syndrome was diagnosed in early 2014 by Mayo Clinic cardiologists. He had presented again to our hospital in Northwestern Wisconsin in February 2015 with new onset recurrent pleuritic chest pain. Earlier in June 2014, the patient was evaluated for left-sided retrosternal chest pain associated with elevated cardiac Troponin I at 0.53 ng/mL (0.0–0.12), elevated CRP at 5.07 mg/l (<3), with EKG showing sinus tachycardia (103 beats per minute), and new nonspecific ST-T wave changes in both anterior and inferior leads (Figure 1A). A cardiac catheterization the same day revealed normal appearing coronary arteries. He was therefore subsequently diagnosed with pericarditis and received oral Ibuprofen 600 mg three times daily for pain control which did not appear to help and patient soon stopped taking the Ibuprofen, anyways. The EKG repeated five days later no longer demonstrated the ST-T segment changes (Figure 1B). Previous ultrasound examination had demonstrated the typical ADPKD polycystic kidneys in April 2013, the right kidney measuring 22 cm in length and the left kidney measuring 18 cm in length (Figure 2A-C). A computed tomography (CT) scan in early 2013 is shown in Figure 2D. The family history was significant for ADPKD in the mother and several siblings. Indeed, the mother has had two renal transplantations for ESRD.

Following the recurrence of the chest pain in February 2015, he was admitted to the hospital for further evaluation. The chest pain was retrosternal, radiated to the upper neck region and was incrementally progressive in intensity over several hours. The pain was better in the sitting position, but was aggravated when he lay down, and also on deep inspiration. Of note, his EKG this time was unremarkable and did not show any ST-T segment changes. Subsequent review of his presentation and further questioning by nephrology revealed that in addition to the pleuritic nature of the chest pain, it became clear that the patient did in fact experience an associated pink to red intermittent hematemesis for some days prior to presentation in the emergency department. As noted above, he had a similar presentation the year before and was treated then for a presumed pericarditis following a normal coronary angiography at that time. He had also been regular with his outpatient in-center hemodialysis treatments, three times a week.

His medications included intravenous iron sucrose and intravenous darbepoetin given during hemodialysis, pantoprazole 40 mg daily, metoprolol, senna, trazodone, calcium carbonate, hydrocodone with acetaminophen for pain, and polyethylene glycol. Drug allergies included tramadol and calcium acetate.

On physical examination, he was in no acute distress, pale, afebrile, not cyanosed, anicteric, showed no finger clubbing, and there was no peripheral edema. His heart rate was 86 beats per minute, regular rate and rhythm, blood pressure of 166/91 mm Hg, with a pulse oximeter reading of 96% on room air. His chest was clear to auscultation and pericardial rubs were not evident.

Laboratory evaluation revealed Hemoglobin 10.3 g/dl, WBC 5800/µl, and platelet count 177000/µl. Electrolytes were normal except for a HC03 of 21 mEq/l. Serum creatinine was 12.72 mg/dl with a BUN of 52 mg/dl, glucose 90 mg/dl, calcium was 9.4 mg/dl and lipase was 34 U/l. Liver panel was normal with albumin 3.8 g/dl, AST 15 U/l, ALT 10 U/l, and total bilirubin 0.3 mg/dl. Coagulation profile was normal with INR at 1.1 and PTT at 29.6 seconds. Urinalysis showed red cloudy urine with dipstick positive tests for proteinuria, hematuria and glycosuria (unclear significance).

Chest radiograph was unremarkable. Abdominal ultrasound did not reveal any significant finding as findings were unchanged from previous ultrasound while confirming previous right nephrectomy. Abdominal CT scan revealed multiple cysts in the upper pole of the left kidney as well as several scattered low density lesions in the liver. The atypical presentation of the chest pain, taken together with the experience in June 2014 when the chest pain did not respond to Ibuprofen (NSAID) then used to empirically treat for presumed pericarditis, and the associated nausea, vomiting with hematemesis triggered a GI medicine consultation for a plausible upper gastrointestinal condition.

**Figure 3:**
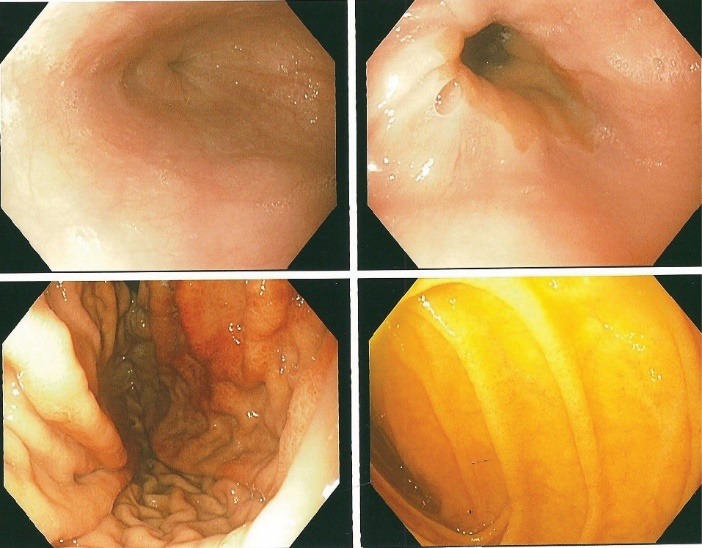
Shows portal hypertensive gastropathy (PHG) changes evident on upper GI endoscopy.

**Figure 4:**
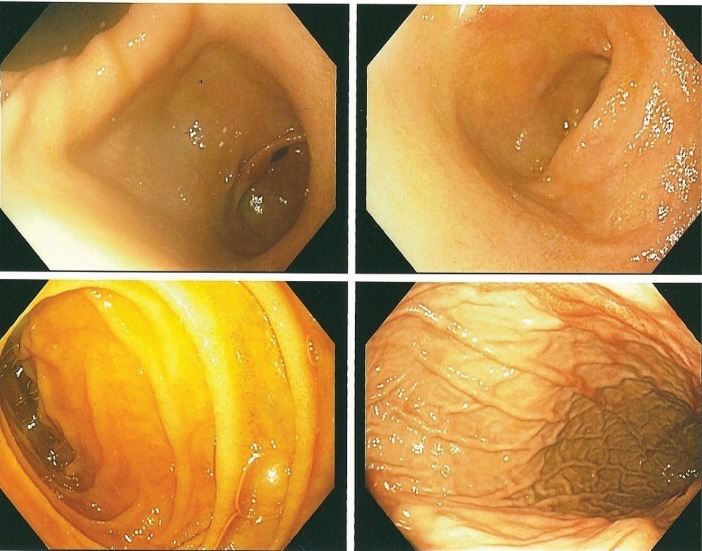
Shows other portal hypertensive gastropathy (PHG) changes evident on upper GI endoscopy

**Figure 5:**
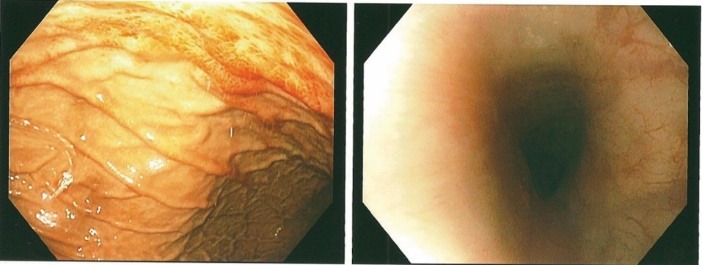
Shows portal hypertensive gastropathy (PHG) more evident on upper GI endoscopy.

Diagnostic esophagogastroduodenoscopy under monitored anesthesia revealed an area of hyperemic looking folds in the gastric body below the gastric cardia with changes consistent with and suggestive of prolapse gastropathy (Figures 3-5). Cold biopsies were obtained from the area as well as random biopsies of the normal appearing gastric antrum. No endoscopic evidence of esophagitis or luminal strictures was evident. Also, no evidence of hiatal hernia, peptic ulceration disease or blood in the upper gastrointestinal tract was observed.

The final impression was that he was suffering from PHG secondary to liver involvement by cysts in ADPKD. Initial treatment included bedrest, adequate hydration, and the doubling of the dose of pantoprazole from 40 mg daily to 40 mg two times daily, together with diet as tolerated. He was discharged after 3 days, with resolution of the chest pain, nausea, vomiting and hematemesis. The following month, in March 2015, the patient successfully received a living related kidney allograft from the father at Mayo Clinic while simultaneously undergoing left sided nephrectomy for severe and recurrent gross hematuria.

## Discussion

Most studies have proposed portal hypertension as the essential underlying factor for PHG. This is attributed to increased mucosal friability and increased levels of mucosal mediators with a background of abnormally dilated vasculature leading to gastric mucosal injury, oxidative stress and impaired healing (2). According to retrospective data analysis, roughly 1 in 7 patients with PHG will develop bleeding (either acute or chronic) attributable to gastropathy (10).

In a setting of ADPKD, hepatic cysts occur in more than 60% of patients (11). Prevalence by magnetic resonance imaging (MRI) scanning in the CRISP study is 58%, 85% and 95% in 15-24 years old, 25-35 years old, 36-46 years old participants respectively (12). These hepatic cysts cause direct mass effect by causing portal vein obstruction, hepatic venous outflow obstruction, bile duct and IVC compression. Also, IGF-1 receptors, oestrogen receptors and GH receptors are expressed in the epithelium lining these hepatic cysts, thus, oestrogen and IGF-1 stimulate hepatic cyst derived cell proliferation (13).

These processes described above if chronic, have been postulated to induce hepatic fibrosis causing portal hypertension in ADPKD and a review of the literature unearthed a case report of hepatic fibrosis associated with ADPKD. Also, bilateral nephrectomy in patients with massively enlarged livers in ADPKD may cause portal hypertension (14,15). Furthermore, acute hepatic vein thrombosis and Budd-Chiari syndrome have been reported after uni- or bilateral nephrectomy in ADPKD patients (16).

From the foregoing, it remains unclear to what extent the antecedent right-sided uninephrectomy in our patient carried out in April 2013 predisposed him to the recurrence of chest pain a year later in June 2014 with the appearance of symptomatic PHG (14-16). Moreover, the impact of the subsequent left nephrectomy at the same time as the kidney transplantation in March 2015 is yet to be fully evident on the presentation of PHG in our patient (14-16). There is also nothing in the literature to suggest that kidney transplantation in ADPKD has any effect on PHG symptomatology. Notably, our patient as at July 2015, has continued to do well, otherwise asymptomatic, and not needing to take any proton pump inhibitors while maintaining a stable renal allograft serum creatinine of 1.5 mg/dl, suggesting that kidney (re)transplantation may have ameliorated PHG in our patient.

In summary, June 2014, our patient presented with sub-sternal chest pain, sinus tachycardia, elevated cardiac enzymes with a normal coronary angiogram and was misdiagnosed as pericarditis. At subsequent presentation with chest pain again in February 2015, the clear association with hematemesis raised the plausibility of a gastrointestinal aetiology for the chest pain. Esophagogastroduodenoscopy revealed changes of PHG (Figures 3-5). Our patient had therefore developed upper GIB secondary to PHG (1-4,10,11). This may have been fuelled or triggered by the preceding therapeutic right-sided nephrectomy which was carried out in April 2013 for life threatening recurrent gross hematuria (14-16). Even though chest pain in ESRD with ADPKD could be due to uremic pericarditis, coronary artery disease, referred pain and rarely pericardial cysts, in patients with non-response to NSAIDs, a high index of suspicion for PHG as the cause of the chest pain must be entertained (1,5-9). Diagnosis is made by esophagogastroduodenoscopy. Initial management usually consists of adequate hydration and maximal doses of a proton pump inhibitor. Thereafter, non-selective β-blockers represent the mainstay of therapy for chronic bleeding, while somatostatin and vasopressin and their derivatives may be used in conjunction with supportive measures for acute bleeding and salvage therapy with trans-jugular intrahepatic portosystemic shunt or rarely surgical shunt is appropriate when medical management fails (2,17,18). The role of endoscopic therapy for PHG is controversial, and whereas balloon-occluded retrograde trans-venous obliteration presents an alternative approach for the management of PHG, liver transplantation should be considered as a final resort in cases of refractory bleeding due to PHG (2,17,18).

## Conclusion

Finally, we propose that PHG should be kept in the differential diagnosis of chest pain, pleuritic or non-pleuritic, with or without gastrointestinal symptoms, in patients with ADPKD, with or without renal failure. Moreover, the importance of a detailed medical history in the management of these patients cannot be over emphasized (9).

## Authors’ contribution

MACO; conception, design, acquisition of data, data analysis, interpretation of data, drafting the article and final approval of manuscript. NA; critical revising for important intellectual content, design, final approval of manuscript. JA; literature review, final approval of manuscript. CO; literature review, interpretation of data and final approval of manuscript. FO; literature review, interpretation of data and final approval of manuscript.

## Conflicts of interest

The authors report no conflicts of interest. The authors alone are responsible for the content and writing of the article.

## Ethical considerations

Ethical issues (including plagiarism, data fabrication, double publication) have been completely observed by the authors.

## Funding/Support

None.
